# Wheat end-use quality: State of art, genetics, genomics-assisted improvement, future challenges, and opportunities

**DOI:** 10.3389/fgene.2022.1032601

**Published:** 2023-01-05

**Authors:** Madhav Subedi, Bikash Ghimire, John White Bagwell, James W. Buck, Mohamed Mergoum

**Affiliations:** ^1^ Institute of Plant Breeding, Genetics and Genomics, University of Georgia, Griffin Campus, Griffin, GA, United States; ^2^ Department of Plant Pathology, University of Georgia, Griffin Campus, Griffin, GA, United States; ^3^ Department of Crop and Soil Sciences, University of Georgia, Griffin Campus, Griffin, GA, United States

**Keywords:** end-use quality, genomics-assisted breeding, quantitative trait locus mapping, marker-assisted selection, genomic selection, candidate gene approach, translational genomics, genome-wide association study

## Abstract

Wheat is the most important source of food, feed, and nutrition for humans and livestock around the world. The expanding population has increasing demands for various wheat products with different quality attributes requiring the development of wheat cultivars that fulfills specific demands of end-users including millers and bakers in the international market. Therefore, wheat breeding programs continually strive to meet these quality standards by screening their improved breeding lines every year. However, the direct measurement of various end-use quality traits such as milling and baking qualities requires a large quantity of grain, traits-specific expensive instruments, time, and an expert workforce which limits the screening process. With the advancement of sequencing technologies, the study of the entire plant genome is possible, and genetic mapping techniques such as quantitative trait locus mapping and genome-wide association studies have enabled researchers to identify loci/genes associated with various end-use quality traits in wheat. Modern breeding techniques such as marker-assisted selection and genomic selection allow the utilization of these genomic resources for the prediction of quality attributes with high accuracy and efficiency which speeds up crop improvement and cultivar development endeavors. In addition, the candidate gene approach through functional as well as comparative genomics has facilitated the translation of the genomic information from several crop species including wild relatives to wheat. This review discusses the various end-use quality traits of wheat, their genetic control mechanisms, the use of genetics and genomics approaches for their improvement, and future challenges and opportunities for wheat breeding.

## 1 Introduction

Wheat is a major cereal crop due to its production and utilization throughout the world. Wheat has been the major source of energy (carbohydrate), protein, and dietary fiber for humankind (Ma et al., 2013). The wheat grain consists of 8%–20% protein and the carbohydrates make up 85% (w/w) of grain, most of which is starch (Anjum et al., 2007; Shewry and Hey, 2015; Nigro et al., 2019). Thus, the nutrition and food security of the world relies on the quantity and quality of wheat being produced worldwide. However, the demand for wheat consumption still surpasses its productivity and there is an urgent need to increase food production by nearly 60% to feed the ever-increasing human population that is predicted to reach 9 billion by 2050 (Zhang et al., 2014; Varshney et al., 2015). Nearly 821 million people, which accounts for one in nine people in the world, suffered from hunger in 2018 (WHO, 2018) further emphasizing the importance of wheat in alleviating hunger. Increased food production is also challenged by decreasing cropland area, finite resources, and the impacts of climate change on overall crop performance.

Wheat breeding programs throughout the world face a common challenge to maintain or improve agronomic performance while simultaneously improving quality traits to fulfill the needs of the diverse international market and end-users including growers, millers, bakers, and consumers (Gale, 2005; Huang et al., 2006; Echeverry-Solarte et al., 2015). To increase the grain yield, researchers have focused on various yield components (grain weight, grain length, spike length, kernel number/spike, spike number/unit area etc.), its correlated trait (plant height, chlorophyll content etc.) and resistance to biotic and abiotic stresses including heat tolerance, and resistance to rusts and *Fusarium* head blight (Zhang et al., 2009; Hanif, 2016; Sun et al., 2017; Kuzay, 2019; Pinto et al., 2019; Sapkota et al., 2019; Yang et al., 2019; Kumar et al., 2007; Ghimire et al., 2020; Liu et al., 2020; Pradhan et al., 2020; Zhang et al., 2020; Ghimire et al., 2022). Consequently, significant progress has been achieved in the past 50 years as wheat yield worldwide increased from 1.4 MT/ha in 1970 to 3.5 MT/ha in 2019 (FAO, 2021).

Overall improvement in the end-use quality of wheat is an inherently complex breeding objective since it is determined by the combination of many component quality traits that are underpinned by diverse metabolic pathways (Mann et al., 2009). Many of these are also correlated with each other which further adds the complexity (Prasad et al., 2003; Mann et al., 2009; Li J et al., 2012). Besides this, the selection of optimum quality trait(s) targeting one user group often comes as a tradeoff for others (Echeverry-Solarte et al., 2015). For example, in North America and Western Europe, bread is made from wheat varieties that produce strongly elastic dough with some extensibility. However, these varieties may not be suitable for making other wheat products such as cookies (biscuits) which are made from highly extensible dough (Payne, 1983). Similarly, chapatti and noodles consumed in South Asia are made from wheat varieties with intermediate properties between the two extremes and pizza and bagels are made from wheat with high gluten strength (Lindgren and Simsek, 2016). Therefore, wheat breeders have to make targets of developing wheat varieties with quality parameters that meet particular demands from millers and consumers (Prasad et al., 2003; Mann et al., 2009).

Through the 19th century, wheat quality essentially meant bread quality to people (Kiszonas and Morris, 2017). In the past few decades, people are more conscious of their dietary habits and have developed a preference for different wheat products with specific quality attributes (Peña, 2007). The development of such a wide range of products was possible due to the studies on the functional and molecular genetic basis of wheat quality particularly wheat protein and its subunits during the 20th century as summarized in several reviews (Payne et al., 1982; Payne, 1987; Weegels et al., 1996; Shewry et al., 1997). The wide range of wheat products creates a broad spectrum of performance specifications that are determined by different end-use quality traits, their genetic and environmental factors and their complex interactions. Improvement in end-use quality of wheat depends will depend on our understanding of all these components, therefore, further studies on the end-use quality of wheat are critical.

Since the turn of the 21st century, research has been carried out in diverse fields including QTL mapping, association mapping and marker-assisted selection (Patil et al., 2009; Reif et al., 2011). There is also diversity in the traits being studied such as starch, grain hardness, flour color, milling and baking quality that has helped to discover many attributes that define the quality of wheat (Zanetti et al., 2001; Sourdille et al., 2003; Kuchel et al., 2006; McCartney et al., 2006). Based on the results of these studies, a few reviews were published that summarized the QTLs/genes and their diagnostic markers to be used for marker-assisted breeding of wheat end-use quality (Gale, 2005; Liu et al., 2012). More recently, studies on genomic selection and translational genomics for end-use quality traits are being carried out which still warrants exhaustive efforts across wheat breeding programs to achieve satisfactory results as is the case for grain yield or disease resistance (Kristensen et al., 2019; Nigro et al., 2019; Aoun et al., 2021). There are limited reviews that capture these modern genetic and genomic studies on diverse end-use quality traits. This review summarizes the key genetic and genomic findings made using different genomic tools on various important end-use quality traits and also points out the current challenges and future opportunities for such studies in wheat.

## 2 Wheat classes and their end-use products

Wheat grown throughout the US are classified into five different classes based on the grain color (red and white wheat), texture (hard and soft wheat), and growth habit (winter and spring wheat) (Clark and Bayles, 1935; https://www.uswheat.org/) thus, categorizing them as hard red winter wheat (HRWW), hard red spring wheat (HRSW), soft red winter wheat (SRWW), soft white wheat (SWW), and hard white wheat (HWW). These classes of wheat also differ based on the products that can be made from them (Clark and Bayles, 1935). For instance, HRWW is suitable for making flat bread, hard rolls, hearth bread, croissants, and all-purpose flour because of its excellent milling and baking characteristics (Chang and Chambers Iv, 1992; https://www.uswheat.org/). The HRSW with high protein content is also referred to as “aristocrat of wheat” for designing wheat products like rolls, bagels, croissants, hearth bread, and pizza crust. SRWW with low protein content is used for making cookies, cakes, crackers, pretzels, and pastries (Faridi et al., 1994). Similarly, SWW also has low protein content and is used for making the best quality pastry, cakes, and other confectionary products. HWW is used for making Asian noodles, pan, and flatbreads (Chang and Chambers Iv, 1992). Durum wheat is the hardest of all kinds of wheat with high protein content and is used for making pasta and couscous (Dick and Youngs, 1988).

## 3 Major end-use quality traits

There are multiple complex traits to consider for defining the quality of wheat importance to wheat producers, end-users, and breeders (McCartney et al., 2006). These include traits related to grain characteristics (protein content, color, weight, grain hardness/texture), milling properties (flour yield, protein content, moisture content, ash content), flour and dough properties (starch content, falling number, gluten characteristics, dough rheology) and baking qualities (loaf height, volume and texture, elongation, mixing time, cookie diameter, baking score) (Mohler et al., 2014; Guzmán and Alvarez, 2016; Hayes et al., 2017; Naraghi et al., 2019). Above all, grain protein content (GPC) has been a focus for both plant breeders and end-users since it directly affects nutritional value, and dough rheological and baking properties (Joppa et al., 1997; Alamri et al., 2009; Brevis et al., 2010). GPC is a critical marketing characteristic and it influences the quality performance of wheat end products in general, including pasta and bread (Mesfin et al., 1999; Distelfeld et al., 2006; Brevis et al., 2010; Naraghi et al., 2019) and it is used as one of the parameters for wheat classification (Tu and Li, 2020). The price of wheat is also determined based on the GPC where wheat with higher GPC is valued more than lower GPC (Farquharson, 2006). Wheat GPC should usually be above 12.5% to be used in bread making (Turner et al., 2004).

Wheat endosperm harbors the majority of the grain content (Osborne, 1907). Gluten protein is a rubbery mass left behind when the starch granules and water-soluble constituents of wheat dough are removed (Wieser, 2007). The quality and quantity of gluten protein is an important trait to be considered for wheat breeding since it determines the baking quality of wheat dough by conferring its viscosity, cohesivity, elasticity, and water absorption capacity (Wieser, 2007). As the protein quality increases, the dough strength, firmness, and stability and cooked weight also increase (Payne, 1987; Dick and Youngs, 1988). Wheat gluten has high glutamine and proline amino acid content (Wieser, 2007). Based on the molecular size in dissociating solvents, wheat endosperm gluten can be categorized into two storage proteins, gliadin and glutenin (Huebner, 1970). The gliadins are small with no disulfide-bonded subunit structure and are soluble in aqueous alcohol, whereas, the glutenins are large, heterogeneous molecules connected by disulfide bonds that are insoluble in aqueous alcohol (Osborne, 1907; Wall, 1979; D’Ovidio and Masci 2004; Kumar et al., 2013). Based on gel electrophoresis, the glutenin subunits can be further divided into predominant low-molecular-weight glutenin subunits (LMW-GS) and high-molecular-weight glutenin subunits (HMW-GS), whereas, gliadin can be separated into four groups (α, β, γ, and ω) (Wall, 1979; D’Ovidio and Masci, 2004). Hydrated glutenin is cohesive and elastic which provides strength and elasticity to dough, whereas, hydrated gliadin is less elastic and cohesive than glutenin and provides dough viscosity and extensibility (Payne et al., 1984; Wieser, 2007; Kumar et al., 2013). Wieser (2007) defines gliadins as a “plasticizer or solvent for glutenins.” Gluten strength can be measured by the Sodium dodecyl sulfate (SDS)-microsedimentation test or sedimentation volume (Kumar et al., 2013).

Grain hardness or texture is a fundamental basis for differentiating wheat market class and trade worldwide (Guzmán and Alvarez 2016). The durum wheat grains are classified as very hard and used for making pasta and couscous whereas the common wheat grains are classified as hard and soft and used for making bread, cakes, noodles, and cookies (Giroux and Morris 1997; Bushuk, 1998). Grain hardness also has a profound effect on the milling, baking, and end-use qualities of wheat (Giroux and Morris 1997). The most common methods for grain texture measurement are Particle Size Index (PSI), Near-Infrared Reflectance (NIR), and the Single Kernel Characterization System (SKCS) (Morris, 2002). The hardness or texture of wheat grains is molecularly determined by two puroindoline proteins, Pina and Pinb, where the grain texture is considered soft when both proteins are functional. However, when one is absent or mutated, the texture is hard, and when both proteins are absent as in the case of durum wheat, the texture is very hard (Morris, 2002).

Starch content and its pasting property also significantly influence wheat end-use products. Starch comprises about 70% of the endosperm dry weight, and it affects grain weight and quality as well as the capacity of plant sink tissues to accept and convert photoassimilates (Dale and Housley, 1986; Kumar et al., 2018; Zi et al., 2018). The starch pasting property has been found to affect the texture and quality of end-use products (Blazek and Copeland 2008). The reserved starch in the plant is comprised mainly of two macromolecules, amylose (22%–35%) and amylopectin (68%–75%) (Nakamura et al., 1995). Amylose in wheat grain is synthesized by granule-bound starch synthase (GBSSI), or waxy protein (Guzmán et al., 2012a). Wheat with reduced amylose is referred to as “partial waxy,” and wheat with no amylose is referred to as “waxy” (Graybosch, 1998). Absence of amylose, i.e., 100% amylopectin, in waxy wheat grains is supposed to help reduce the staling of flour products, especially bread, and keep baked goods fresh for longer periods (Zi et al., 2018). However, waxy wheat lines have reduced grain yield and increased amylose content has also been associated with nutritional and physiological effects (Nakamura et al., 1995; Blazek and Copeland, 2008; Zi et al., 2018). Partial waxy wheat is considered useful for producing high quality thick white noodles such as udon noodle used in Japanese cuisine (Zhang et al., 2022). Non-waxy and waxy starches can be differentiated by staining with iodine, where non-waxy starch stains blue-black and waxy starch stains red-brown (Nakamura et al., 1995). The relative amounts of amylose and amylopectin determine the physical and chemical properties of starch, such as pasting, gelation, and gelatinization, which determine the quality of end-product (Fredriksson et al., 1998; Guzmán and Alvarez 2016). There are various methods for measuring starch, amylose, and amylopectin, including the dual wavelength iodine binding method, differential scanning calorimetry (DSC), and high-performance size-exclusion chromatography (HPSEC) (Zhu et al., 2008).

The end-use quality of wheat is also determined by dough rheological properties and falling numbers. The rheological properties of wheat are estimated by water absorption, dough development time, dough stability, maximum dough resistance, dough extensibility, and flour paste viscosity (Guo J. et al., 2020). Dough rheology can be measured by using farinograph, extensograph, and alveograph instruments (AbuHammad et al., 2012). These dough rheological properties along with other traits such as flour protein content (FPC), particle size, loaf volume, and crumb score can be used for estimating the baking quality of wheat (Kuchel et al., 2006). During germination, starch in the wheat grain needs to be converted to simple sugars to feed the embryo. Alpha-amylase is one of the primary enzymes responsible for the starch degradation causing sprouting of the grain (Newberry et al., 2018). Such alpha-amylase activity is only desirable if the grain has been planted (Thomason et al., 2019). However, some wheat genotypes are characterized by an excessive level of alpha-amylase from the grain development stage to harvest causing pre-harvest sprouting (PHS). PHS results in lower yield and affects the end-use quality of wheat such as dough softening, sticky bread crumb, and problems while slicing bread (Mohler et al., 2014; Newberry et al., 2018). The falling number determines the effect of alpha-amylase activity on damaging starch by examining the starch pasting property (Mohler et al., 2014). Increased level of the alpha-amylase reduces the value of falling number (Thomason et al., 2019).

## 4 Breeding methods for end-use quality traits of wheat

### 4.1 Direct phenotypic selection

To select individual plants with superior quality traits, wheat breeders used to grow multiple individuals/lines in different environments, harvest grain, process it, and evaluate it for different milling and baking quality parameters. All these wheat qualities had to be evaluated precisely using appropriate instruments under lab conditions which makes this procedure challenging because it is expensive, time-consuming, labor-intensive, and typically requires large seed samples (Blanco et al., 1996; Prasad et al., 2003; Naraghi et al., 2019; Yang et al., 2020). Analyses requires time, which often leads to increase in 1 year for the breeding program or passing wheat lines with undesirable quality alleles into the next growing season (Sandhu et al., 2021). Moreover, if these evaluations are carried out later in the breeding program, the developed wheat lines could end up with poor end-use quality and be discarded leading to waste of resources as the primary focus was centered on improving other traits such as grain yield and disease resistance (Naraghi et al., 2019). Therefore the direct selection method for end-use qualities of wheat has been more complicated for wheat breeders (Yang et al., 2020). Traits like GPC and FPC are also influenced by the genotype by environment (G×E) interaction leading to low heritability (Blanco et al., 1996; Groos et al., 2003; Prasad et al., 2003). As a result, accurate assessment of these traits in the breeding programs is quite challenging which makes the selection process even more complicated.

### 4.2 Modern approaches: Genomics-assisted breeding (GAB) and translational genomics

**FIGURE 1:**
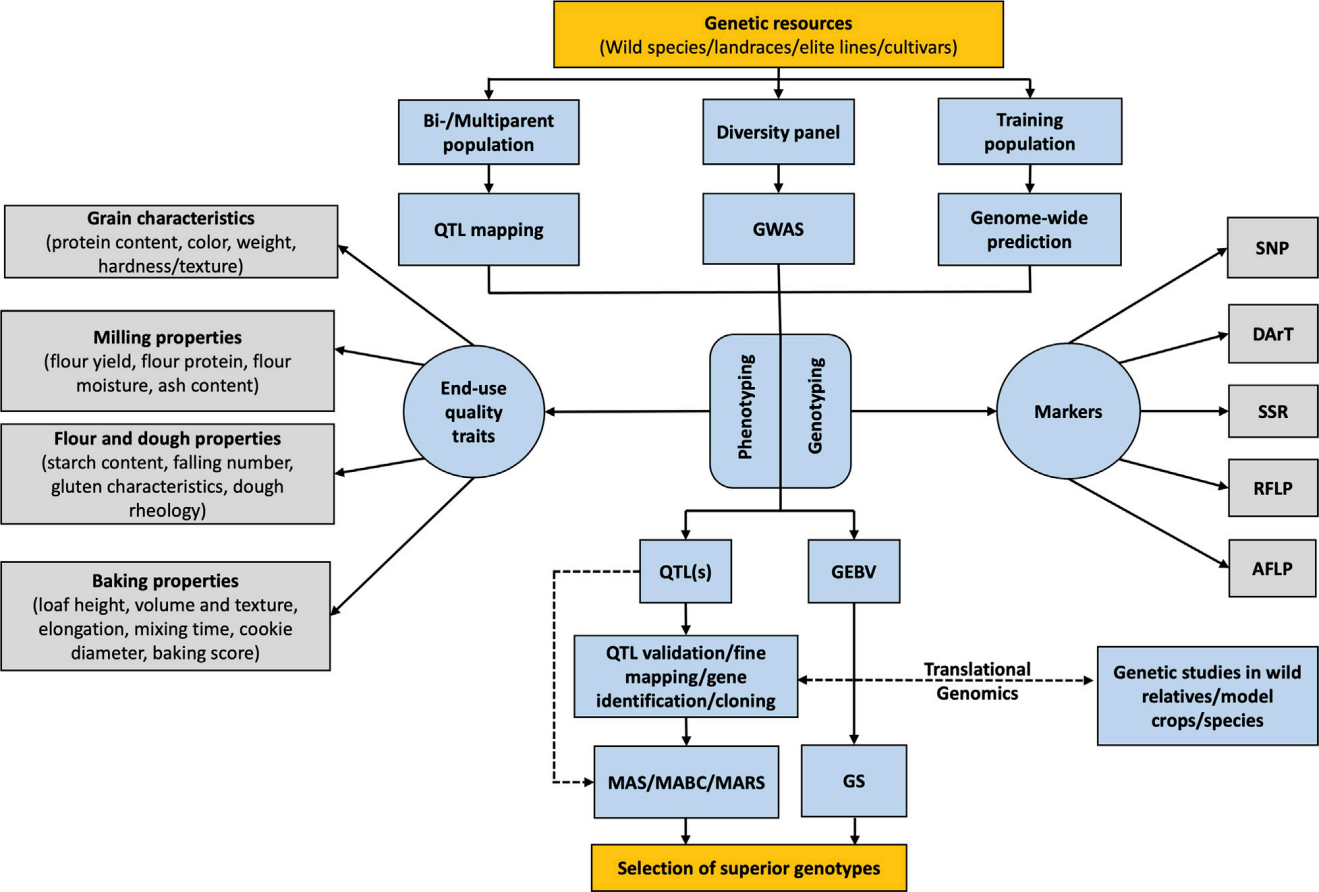
Schematic flow diagram showing the use of genomics-assisted breeding and translational genomics for improvement of end-use quality traits in wheat breeding program.

Genome-based technologies are an important means of breeding for improved wheat quality (Kiszonas and Morris, 2017). The advancement in sequencing technologies and their utilization for genomic research brought increased precision and efficiency to crop breeding (Varshney et al., 2005; Varshney et al., 2015). Various kinds of molecular markers such as restriction fragment length polymorphism (RFLP), simple sequence repeats (SSR), and single nucleotide polymorphism (SNP) are being used for genetic mapping to identify quantitative trait loci (QTL) and functional markers related to the genes of interest (Figure 1; Table 1) (Groos et al., 2003; Prasad et al., 2003; Rimbert et al., 2018). These markers can be exploited for marker-assisted selection (MAS) and genomic selection (GS) which allow screening for superior end-use quality traits earlier in the breeding program (Figure 1; Tables 2, 3) (Naraghi et al., 2019). These approaches are convenient and faster than the traditional selection method. This allowed the transition from solely phenotype-based traditional selection to a genotype-linked phenotype-assisted selection of superior lines for different plant species (D’hoop et al., 2014; dos Santos et al., 2016; Proietti et al., 2018; Bhattarai and Subudhi, 2019). However, given the complex inheritence nature of these quantitative traits and the significant effects of the environment and the G×E interaction for these traits, phenotypic selection is still required to confirm the efficacity of modern methods including MAS and GS.

**TABLE 1:** Summary of representative QTL identification studies for end-use quality traits in wheat. Phenotypic variance is indicated collectively for all the QTLs or individually for each QTL.

Model species	Population type^a^	Mapping population	End-use quality traits^b^	Marker type^c^	Number of QTLs identified	Genome location	Phenotypic variance explained	References
Durum wheat	RIL	Messapia (cultivar of *T. turgidum* L. subsp. Durum) × MG4343 (wild accession of *T. turgidum* L var *dicoccoides*)	GPC	RFLP	6	4BS, 5AL, 6AS, 6BS, and 7BS	6.0%–23.5% individually	Blanco et al. (1996)
Durum wheat	RICL	LDN (DIC-6B) × LDN durum	GPC	RLFP	1	6BS	66%	Joppa et al. (1997)
Durum wheat	RIL	Messapia (cultivar of *T. turgidum* L. subsp. Durum) × MG4343 (wild accession of *T. turgidum* L var *dicoccoides*)	SV	RFLP	7	1AL, 1B, 3AS, 3BL, 5AL, 6AL, 7BS	36%–64% collectively	Blanco et al. (1998)
Common wheat	RIL	Glupro × Keene, Glupro × Bergen, ND683 × Bergen	GPC	RFLP	1	6BS, 6BL	12.4%–34.6% across three populations	Mesfin et al. (1999)
Common wheat and spelt	RIL	*T. aestivum* L variety “Forno” × *T. spelta* L variety ‘Oberkulmer	FPC, SV, dough properties, GH, TKW	RFLP	FPC: 9; SV: 9; dough properties: 10; GH: 10; TKW:8	FPC: 3B, 4A, 5B, 6B, 7A, 7B, 7D; SV: 1B, 1D, 2A, 3A, 5A, 5B, 5D; dough properties: 1A, 1B, 2A, 2B, 2D, 3A, 3B, 3DL, 4A, 4D, 5A, 5D,7A, 7B, 7D; GH: 2A, 3B, 4A, 4D, 5A, 6B, 7A,7B, 7D; TKW: 1B, 2B, 3B, 5A, 5B, 7B	Protein: 51%; SV: 47%; GH: 54%; TKW: 54%; and dough properties: 39% collectively	Zanetti et al. (2001)
Common wheat	RIL	Renan (variety) × Recital (variety)	GPC, TKW	SSR, RFLP, AFLP	GPC: 10; TKW: 9	GPC:1A, 2A, 3A, 3B,4A, 4D, 5B, 6A, 7A, 7D; TKW: 1D, 2B, 2D, 3A, 5B, 6A, 6D, 7A, 7D	GPC: 4.2%–10.4%; TKW: 4.7%–19.7% individually	Groos et al. (2003)
Common wheat	DH	Courtot (cultivar) × Chinese spring (cultivar)	Protein content GH, Dough strength	SSR, AFLP	Protein content: 2; GH: 3; dough strength: 3	Protein content: 1BL, 6AS; GH: 1AL, 5DS, 6AL; dough strength: 1AL, 3BL, 5DS	Protein content: 6.5%–17.1%; GH: 3.1%–66.9%; dough strength: 9.4%–19.5% individually	Sourdille et al. (2003)
Common wheat	RIL, NIL	RIL: WL711 × PH132 NIL: WL711 × PH132, WL711 × PH133, HD2329 × PH132	GPC	SSR	13	2A, 2B, 2D, 3D, 4A, 6B, 7A, 7D	2.95%–32.44% individually	Prasad et al. (2003)
Common wheat	DH	Trident (cultivar) × Molineux (cultivar)	FPC, Flour color, Flour brightness, dough properties, baking quality	SSR, STS	FPC: 5; flour brightness: 2; flour color: 3; dough properties: 18; baking quality: 4	FPC: 1B, 6A, 6D, 7A, 7D; flour brightness: 1A, 7B; flour color: 1A, 7B; dough properties: 1A, 1B, 2A, 2B, 2D, 3D, 7A, 7D; baking quality: 2A, 3A	FPC: 6%–13%; flour brightness: 10%–22%; flour color: 6%–77%, dough properties:5%–20%, baking quality:5%–13% individually	Kuchel et al. (2006)
Common wheat	DH	AC karma (variety) × 87E03-S2B1 (breeding line)	GPC, FPC, MG, SV, TKW	SSR	GPC: 2; FPC: 3; MG: 18; SV: 3; TKW: 6	GPC: 4D, 7B; FPC: 2D,4D,7B; MG: 1B,1D,3B,4D,5D; SV: 1B,2D,5D; TKW: 2B, 2D, 3B, 4B, 4D, 6A	GPC: 12.6%–32.7%; FPC: 6.6%–28.6%; MG: 6%–55.9%; SV: 8.8%–14.9%; TKW: 3.7%–26.3% individually	Huang et al. (2006)
Common wheat	DH	RL4452 × AC Domain	47 traits including Milling (GPC, FPC, PSI, FY), MG, FG, baking, starch properties, noodle color, and others	SSR	Milling: 12; MG: 24; FG: 9; baking: 14; starch properties: 19; noodle color: 11; others: 10	1A, 1B, 1D,2A, 2B, 3A, 3B, 3D, 4A, 4B, 4D, 5B, 5D, 6A, 6B, 7A, 7B, 7D for all traits combined	GPC: 6.2–29.8; FPC: 6.1%–28.7%; FY: 7.9%–11.9%; PSI:28.2%; MG: 4.4%–42%; FG: 4.6%–35.4%; baking: 7.9%–24.8%; starch properties: 4.1%–54.4%; noodle color: 7.7%–36% individually	McCartney et al. (2006)
Durum wheat	RIL	PDW 233 × Bhalegaon 4 (Landrace)	SV, GPC, MG	SSR, ISSR, SCAR, TRAP	26 main effect QTLs in total from 3 environments	SV: 1B; GPC: 7B; MG: 1A, 1B, 2B, 4B, 7A, 7B	SV: 6.7%–40.66%; GPC: 9.64%; MG: 6.75%–21.32% individually	Patil et al. (2009)
Common wheat	DH	Kurki (cultivar) × Janz (cultivar)	GPC, sponge and dough baking performance, GH	SSR	-	GPC: 1B, 3A, 5A, 5B, 7A; sponge and dough making: 1B,1D,3A, 4D, 5B, 5D, 7A, 7B; GH: 1A, 4D, 5D	-	Mann et al. (2009)
Durum wheat	DH	Rugby (cultivar) × Maier (cultivar)	Gluten strength	DArT, STS, EST-SSR	3	major QTL in 1BS	90% by single major QTL	Kumar et al. (2013)
Common wheat	RIL	WCB414 (elite line) × WCB617(exotic line)	TKW, KVW, GPC, FE, MG	DArT	TKW: 11; KVW: 10; GPC: 11; FE: 6, MG: 31	TKW: 2A, 2B, 2D, 3A, 3B, 4A, 5A, 6B; KVW: 1A, 1B, 2A, 4B, 5B, 6A, 6B, 7B; FE: 1A, 1B, 2B, 3D, 4A, 6A; GPC: 1A, 1B, 2B, 2D, 3D, 4B, 5B, 6B, 7B; MG: 2B, 3A, 6A, 6B, 7A, 7B, 7D	TKW: 6.2%–17.1%; KVW: 6.7%–22.5%; FE: 4.9–19; GPC: 4.7–16.9; MG: 5.3–19.9 individually	Echeverry-Solarte et al. (2015)
Common wheat	DH	Yumechikara (HRWW variety) × Kitahonami (SRWW variety)	GPC, FPC	SSR	1	2B	GPC: 32%; FPC: 16.5%	Terasawa et al. (2016)
Common wheat	RIL	Shannong01-35 (variety) × Gaocheng9411 (variety)	Pasting property	SNP, DArT, SSR	43 QTLs from 3 environments	1A, 1B, 2B, 2D, 3A, 4A, 4B, 5B, 6A, 6B, 7A	.11%–37.68% individually	Wang et al. (2017)
Common wheat	RIL	Glenn × Traverse (both hard red spring wheat cultivars)	GPC, FE and MG, Baking properties	SNP	GPC: additive QTL-11, digenic epistatic QTL-18; FE and MG: additive-32, digenic epistatic-51; baking properties: additive QTL-31, digenic epistatic-15	GPC (additive QTL): 1A, 1B, 2A, 2B, 3A, 3B, 4B, 5B, 7A; FE and MG (additive QTL): 1D, 2B, 3D, 5A, 6A, 6D; baking properties (additive QTL): all chromosomes except 1A, 2B, 3D, 6A	GPC: 6.5%–20%; FE and MG: 1%–24%, and baking properties: 2%–28% individually for additive QTLs	Naraghi et al. (2019)
Durum wheat	Diversity panel	7 *T. turgidum* subspecies*: durum, durum* var *ethiopicum, turanicum, polonicum, turgidum, carthlicum, dicoccum,* and *dicoccoides*	GPC	SNP	11 stable QTLs in at least 3 environments out of 7 tested environments	2BS, 3AL, 3BL,4AS, 4BL, 5AS, 5BL, 6BL, 7AS, 7BL	5.1%–8.7% individually across all environments	Nigro et al. (2019)
Common wheat	RIL	ND 705 (elite line) × PI 414566 (exotic line)	GPC, KH, FE	SNP	GPC: 14; KH: 7; FE: 11	GPC: 1A, 1B, 2A, 4B, 4D, 5A, 5B, 6D, 7A, 7B, 7D; KH: 1A, 1B, 4B, 5A, 7A; FE: 1A, 2A, 3A, 3B, 4D, 5A, 5B, 5D, 7D	GPC: 5.6%–24.9%; KH: 7.4%–21.3%; FE: 6.3%–12.6% individually	Kumar A et al. (2019)
Common wheat	Diversity panel	SRWW	FY, FPC, GH, FPC, SRC	SN	FY: 3; FPC: 5; SE: 2; SRC: 8	FY: 1B, 2A, 2B; FPC: 5A, 6A, 7A; GH: 4A, 4B; SRC: 1A, 1B, 3A, 4B, 5B, 7D	3.4%–6.0% individually	Gaire et al. (2019)
Common wheat	Diversity panel	Winter wheat	Grain quality traits (GPC, test weight, WGC, SV, FY, TSC), dough rheological properties (DDT, DS and DWA)	SNP	Grain quality traits: 246 QTNs; Dough rheological properties: 86	40 stable QTNs found in 1A, AD, 1D, 2A, 2B, 2D, 3A, 3B, 3D, 4B, 4D, 5A, 5D, 6A, 6B, 6D, 7A, 7B, 7D	40 stable QTNs explained 4.38%–18.6% individually	Yang et al. (2020)
Common wheat	DH	Two DH populations, Yecora Rojo × Ksu106 and Klasic × Ksu105	FG, MG traits	SNP	Total 176 additive QTLs for FG and MG traits in two populations combined	QTLs found in almost all 21 chromosomes in both populations	103 QTLs with PV ≥ 10% individually in all traits combining two populations	Barakat et al. (2020a)
Common wheat	DH	Two DH populations, Yecora Rojo × Ksu106 (YK) and Klasic × Ksu105(KK)	8 traits including FPC, gluten index, FN, AC, test weight, FM	SNP	Total 127 additive QTLs for all traits in two populations combined	QTLs found in almost all 21 chromosomes in both populations	72 QTLs with PV ≥ 10% individually in all traits combining two populations	Barakat et al. (2020b)
Common wheat	RIL	Tainong 18 (cultivar)× Linmai 6 (elite line)	GPC, SV, FG, FN, Starch pasting properties	SNP, DArT, SSR, EST-SSR	GPC: 10; SV: 11; FG: 17, FN: 4; starch pasting: 64	Protein: 1B,3B, 4A, 4B, 4D, 6A; SV: 1A, 1D, 4B, 5B, 5D, 6A, 6D	Protein: 7%–15.5%; SV: 5.3%–18.73%; FG: 5.6%–35%; FN: 8.4–10.7: starch pasting: 6.3%–17.9% individually	Guo et al. (2020a)
Common wheat	Diversity panel	SWW breeding lines and DH	14 traits categorized as grain characteristics, milling traits, flour characteristics and baking quality	SNP	Total 178 MTA including 12 large effect MTA	Large effect QTLs for Grain characteristics: 1B, 2B, 4B, 5A, and 6B; Milling traits: 1B, 1D, 5A, and 6B; Flour characteristics: 1B, 1D and 4A	-	Aoun et al. (2021)

^a^ RIL, recombinant inbred line; RICL, recombinant inbred chromosome line; NIL, near-isogenic lines; DH, doubled haploid.

^b^ GPC, grain protein content; FPC, flour protein content; SV, sedimentation volume; MG, mixograph; FG, farinograph; FN, falling number; GH, grain hardness; PSI, particle size index; FY, flour yield; FE, flour extraction; AC, ash content; FM, flour moisture; TKW, thousand kernel weight; KVW, kernel volume weight; SRC, solvent retention capacity; WGC, wet gluten content; TSC, total starch content; DDT, dough development time; DS, dough stability; DWA, dough water absorption.

^c^ RFLP, restriction fragment length polymorphism; SSR, simple sequence repeat; AFLP, amplified fragment length polymorphism; STS, sequence-tagged site; ISSR, inter simple sequence repeat; SCAR, sequence characterized amplified region; TRAP, target region amplified polymorphism; DArT, diversity array technology; SNP, single nucleotide polymorphism; EST-SSR, expressed sequence tag-derived simple sequence repeat.

**TABLE 2:** An overview of markers used for marker-assisted selection (MAS) of end-use quality traits in wheat.

End-use quality trait^a^	Genes	Allele(s)/subunits	Chromosome	Marker type(s)^b^	References
GPC	*Gpc-B1*	Gene specific	6BS	SSR (*Xucw108*)	Uauy et al. (2006), Vishwakarma et al. (2014), Gautam et al. (2020)
		Linked	6BS	SSR (*Xuhw89*)	Distelfeld et al. (2006), Vishwakarma et al. (2014), Gupta et al. (2022)
HMW-GS	*Glu-A1*	Ax1, AxNull, Ax2*	1AL	AS-PCR, KASP	de Bustos et al. (2001), Ma et al. (2003), Ravel et al. (2020)
	*Glu-B1*	Bx7, Bx8, Bx9, Bx13, Bx14, Bx15, Bx16, Bx17, Bx20, Bx23, Bx7^OE^	1BL	AS-PCR, STS, KASP	Ma et al. (2003), Butow et al. (2004), Lei et al. (2006), Ravel et al. (2020)
	*Glu-D1*	Dx2, Dx5, Dx3, Dy10, Dy12, gene specific	1DL	AS-PCR, KASP	Smith et al. (1994), de Bustos et al. (2001), Ma et al. (2003), Ravel et al. (2020), D’Ovidio and Anderson (1994)
LMW-GS	*Glu-A3*	*a, b, c, d, e, f, g*	1AS	STS, KASP	Wang et al. (2010), Dreisigacker et al. (2020)
	*Glu-B3*	*a, b, c, d, e, f, fg, g, h, i*	1BS	AS-PCR, STS, KASP	Shan et al. (2007), Wang et al. (2009), Dreisigacker et al. (2020)
	*Glu-D3*	*a, b, c, d, e, g, h, i, j, k*	1DS	STS	Zhao et al. (2007), Appelbee et al. (2009)
Grain hardness	*Pina-D1*	*Pina-D1a, Pina-D1b*	5DS	AS-PCR, STS	Gautier et al. (1994), Huang and Brûlé-Babel (2011), Chen et al. (2013), Rai et al. (2019) Qamar et al. (2014)
	*Pinb-D1*	*Pinb-D1a, Pinb-D1b, Pinb-D1c, Pinb-D1d, Pinb-D1e, Pinb-D1p*	5DS	STS, CAPS	Gautier et al. (1994), Tranquilli et al. (1999), Lillemo and Morris, (2000), Huang and Brûlé-Babel (2011), Qamar et al. (2014)
Starch	*Wx-A1*	*Wx-A1a, Wx-A1b, WxA1-c, Wx-A1d, Wx-A1e, Wx-A1f, Wx-A1g, Wx-A1h, Wx-A1i, Wx-A1* null mutant	7AS	SSR, AS-PCR, STS, RFLP, KASP	Vrinten et al. (1999), McLauchlan et al. (2001), Nakamura et al. (2002), Saito et al. (2004), Monari et al. (2005), Shan et al. (2007), Vanzetti et al. (2010), Yamamori and Guzmán (2013), Zhang et al. (2022)
	*Wx-B1*	Null *Wx-B1, Wx-B1a, Wx-B1b, Wx-B1e, Wx-B1*	4AL	SSR, AS-PCR, STS, RFLP	Briney et al. (1998), Vrinten et al. (1999), McLauchlan et al. (2001), Saito et al. (2004), Monari et al. (2005), Shan et al. (2007), Saito et al. (2009), Divashuk et al. (2011)
	*Wx-D1*	*WxD1a, Wx-D1b, Wx-D1*	7DS	AS-PCR, SSR, STS, RFLP	Vrinten et al. (1999), McLauchlan et al. (2001), Nakamura et al. (2002), Saito et al. (2004), Monari et al. (2005)

^a^ GPC, grain protein content; HMW-GS, high molecular weight-glutenin subunit; LMW-GS, low molecular weight-glutenin subunit.

^b^ STS, sequence-tagged site; CAPS, cleaved amplified polymorphic sequences; AS-PCR, allele specific-polymerase chain reaction; RFLP, restriction fragment length polymorphism; SSR, simple sequence repeat; KASP, kompetitive allele specific PCR.

**TABLE 3:** Summary of few representative genome-based prediction studies for end-use quality traits in wheat.

End-use quality traits^a^	Germplasm^b^	Marker^c^	Model(s) used^d^	Accuracy	References
FY, softness, gluten strength, water absorption, FPC, starch properties	Two DH biparental population of SWW of 209 and 174 individuals	DArT, AFLP, RFLP, SSR, STS	Multiple linear regression, Ridge regression, Bayes-C∏	.42–.66	Heffner et al. (2011)
GPC, FPC, test weight, TKW, grain hardness, FY, dough rheology, loaf volume, gluten strength	5,520 advanced lines of common wheat	SNP	PLSR, elastic net, Random Forest	.32–.62	Battenfield et al. (2016)
19 traits total including grain characteristics, milling traits, dough rheology, noodle traits, baking traits	2076 common wheat accessions	SNP	NA	>.5 for many traits	Hayes et al. (2017)
Protein content, dough rheology, baking quality	840 winter wheat lines including inbreds and DHs	DArT	RR-BLUP, W-BLUP	.38–.63	Michel et al. (2018)
Protein content	Two durum wheat panels of 189 and 159 genotypes consisting of varieties and breeding lines	DArT, SNP	RR-BLUP	.40–.46	Rapp et al. (2018)
GPC, alveograph measurements	Durum wheat panel consisting of 170 varieties and advanced breeding lines, and 154 DHs	SNP	Single trait models: RR-BLUP, G-BLUP, Bayes A, Bayes B, Bayesian LASSO, RKHS, Multi-trait models: MT-Bayes A, MT-Matrix, MT-SI	.5–.8 for single trait models	Haile et al. (2018)
FY, alveograph measurements	635 winter wheat breeding lines	SNP	G-BLUP, Bayesian Power Lasso	.50–.79	Kristensen et al. (2019)
14 traits including grain characteristics, milling traits, flour parameters, and baking traits	SWW panel of 666 lines consisting of inbreds, advanced breeding lines, and DHs	SNP	RR-BLUP, Bayes A, Bayes B, Bayes Lasso, Bayes C, Random Forest, SVM, MLP, CNN	average .58–.63 for all traits and models	Sandhu et al. (2021)
GPC, FPC, FY, dough rheology, bake absorption, bake mixing time, loaf volume, baking score	462 advanced breeding lines of winter wheat	SNP	G-BLUP	.25–.55	Zhang-Biehn et al. (2021)
14 traits including grain characteristics, milling traits, flour parameters, and baking traits	SWW panel of 672 lines consisting of breeding lines, cultivars, and DHs	SNP	G-BLUP	∼.35–.65	Aoun et al. (2021)

^a^ GPC, grain protein content; FPC, flour protein content; FY, flour yield; FPC, flour protein content; TKW, thousand kernel weight.

^b^ DHs, double haploids; SWW, soft white wheat.

^c^ SNP, single nucleotide polymorphism; STS, sequence-tagged site; DArT, diversity array technology; RFLP, restriction fragment length polymorphism; AFLP, amplified fragment length polymorphism.

^d^ PLSR, partial least squares regression; SVM, support vector machine; MLP, multilayer perceptron; CNN, convolutional neural network; G-BLUP, genomic best linear unbiased prediction model; W-BLUP, weighted genomic best linear unbiased prediction; RR-BLUP, ridge regression best linear unbiased prediction.

The availability of plant genome sequence helps in the identification of candidate genes that are linked to available markers. However, initial sequencing was expensive and conducted by fewer labs which resulted in sequenced genomes of only a few model plant species including *Arabidopsis* (The Arabidopsis Genome Initiative, 2000), rice (International Rice Genome Sequencing Project and Sasaki, 2005), and maize (Schnable et al., 2009). Therefore, a lot of earlier genomic studies were conducted in these model plants and their findings were eventually transferred to other crop species which is now referred as the translational genomics apporach (Varshney et al., 2015). Rice was the first plant species to be used as a model cereal crop due to its small genome size and the availability of a well-mapped and characterized genome compared to other cereals like maize and wheat (Eckardt, 2000). With the revolutionization in next-generation sequencing (NGS) technologies such as Illumina and Nanopore sequencing, genome sequencing is much more accessible and affordable than ever before. Currrently, it is possible to genotype 10,000 lines at the same cost associated to phenotyping 1,000 wheat lines for end-use quality traits (Battenfield et al., 2016). Various molecular breeding tools including, but not limited to, biparental linkage mapping, genome-wide association studies (GWAS), MAS, and GS have been developed and are being extensively utilized for identifying functional loci/genes and for predicting the genomic/breeding value of individuals (Figure 1). This genomic information is then utilized for crop improvement and is referred to as genomics-assisted breeding (GAB). The molecular breeding tools used in GAB for wheat quality are discussed later in this chapter.

GAB facilitates the generation of an integrated database of genetic and genomic resources of many crops that can be used for translational genomics (Varshney et al., 2015). Translational genomics utilizes the scope of comparative, functional, and evolutionary genomics for finding relevant information from model species (Kang et al., 2016). Candidate gene approach (CGA) is one of the important tools of translational genomics. CGA assumes that genes within the model and target species could govern similar functions or influence the same trait (Salentijn et al., 2007). These genes could be either functional candidate genes with an identified or predicted function or positional candidate genes that are co-localized with a trait locus (Pflieger et al., 2001). As an example, the flowering time trait of many angiosperm species is functionally related to *Flowering Locus T (FT)* gene homologs (Pin and Nilsson, 2012; Subedi et al., 2021) and can be utilized for the study of the trait in any other flowering species. Positional candidate genes used for CGA can be genes identified through QTL mapping, marker-trait association, or syntenic regions between different genomes (Salentijn et al., 2007). Once the candidate gene has been identified, it can be translated to the target crop and validated through reverse genetic approaches (Figure 1).

There are several ways for validating orthologous genes in the target crop including the Targeting induced Local Lesion in genomes (TILLING) (Chen et al., 2012) and Clustered Regularly Interspaced Short Palindromic Repeats (CRISPR) (Liang et al., 2017). TILLING by sequencing (TbyS) was successfully carried out for translation of genes in Mung bean from medicago, cowpea, and soybean (Tsai et al., 2011). CRISPR gene editing techniques have become extremely popular and are widely used in various crop species. There are many reviews available on the use of CRISPR gene editing technique for crop improvement (e.g., Bortesi and Fischer, 2015; Song et al., 2016; Arora and Narula, 2017). One of the concerns related to CRISPR technology is the presence of transgene after editing and the possibility of off-target mutations which can face regulatory restrictions (Liang et al., 2017). However, methods have been developed and deployed in wheat to create mutants with no detectable transgenes (Liang et al., 2017). This eliminates the need for laborious and time-consuming backcrossing steps to segregate away the CRISPR/Cas9 cassette. Therefore, researchers can select the validation methods according to their interest and resource availability. To complement the validation step of translational genomics, fine-mapping can be done in a population segregating for the trait and associating polymorphism within the candidate gene to the phenotypic variation (Salentijn et al., 2007). Since plant genome sequencing has become fairly routine, the goal now is to sequence every crop species and apply GAB techniques for crop improvement. In this context, there are a plethora of genomic resources from many different crops that can be utilized for translational genomics.

## 5 Genetic and genomic studies on wheat end-use quality traits: An overview

Genetic studies of quality traits are complex due to the polygenic nature of the traits and high G×E interaction (Zanetti et al., 2001; Prasad et al., 2003; Huang et al., 2006). In addition, due to the polyploidy nature of common wheat, recessive mutation phenotypic effects are masked by the effective homoeoloci of other genomes creating an extra challenge in intrachromosomal mapping (Kuspira and Unrau, 1957; Chao et al., 1989). As a result, researchers have focused on increasing marker density to correctly identify loci associated with a certain phenotype. The use of molecular markers for the assessment of quality traits has been of interest since the late 1990s (Galande et al., 2001). Researchers are also selecting genotyping methods that allow better genome coverage and can identify a higher number of genetic variations.

Initial research for end-use quality traits was often carried out using RFLP markers that are unlimited in number, generally codominant, and able to recognize individual loci, thus being effective for the hexaploid genome of common wheat (Chao et al., 1989; Blanco et al., 1996; Joppa et al., 1997). However, RFLP markers were found to show a low level of polymorphism in wheat, especially when studying progenies of closely related genotypes (Devos et al., 1992). Later, microsatellites also called SSR, became more popular since they show a higher level of polymorphism than RFLP markers (Huang et al., 2006). SSR markers were used for linkage mapping and QTL identification for end-use quality traits (Table 1) (Prasad et al., 2003; Sourdille et al., 2003; Kuchel et al., 2006). A combination of RFLP, SSR, and Amplified Fragment Length Polymorphism (AFLP) was also used to increase the marker density in maps (Table 1) (Sourdille et al., 2003; Mohler et al., 2014). With the advancement in sequencing technologies, the use of SNP has been prioritized since they are abundant and uniformly distributed in a genome providing high genomic resolution (Gupta et al., 2008; Kang et al., 2016; Wang et al., 2017; Naraghi et al., 2019; Guo Y. et al., 2020). The availability of high throughput sequencing platform for SNPs makes it a preferred genotyping method in general. Genotyping-by-sequencing (GBS) has been a popular genotyping method that allows capturing SNPs through a reduced representation of the genome (Poland et al., 2012). A few of the representative QTL mapping studies utilizing various genotyping methods for end-use quality traits in wheat are summarized in Table 1.

QTLs are identified either by QTL mapping in a biparental mapping population or by association mapping (Beló et al., 2008). The majority of prior research work in wheat quality traits was concentrated on QTL mapping using different mapping populations like F2 generation, recombinant inbred lines (RILs), doubled-haploids (DHs), and near-isogenic lines (NILs) (Table 1). Recently, GWAS has also been a popular tool for the genetic study of complex polygenic traits in species such as wheat, rice, maize, and barley because of many advantages over QTL mapping (Yang et al., 2020). Since GWAS is carried out in a diversity panel, it has higher allelic diversity and higher resolution as a result of genetic recombination events (Korte and Farlow, 2013). GWAS also allows the detection of quantitative trait nucleotides (QTNs) with small effects associated with complex traits (Yang et al., 2020). However, GWAS has less power and requires a larger population to detect an association compared to QTL mapping. On the positive side, GWAS can be used for the selection of parents for subsequent QTL analysis thus complementing each other (Korte and Farlow, 2013). A few representative GWAS studies for end-use quality traits are summarized in Table 1. With the assembly being available, research can be concentrated on the identification of candidate genes using QTL mapping and association studies (Li and Yang, 2017).

The generation of wheat genome assembly has been a great milestone for improving wheat end-use quality traits. A reference genome has allowed us to physically map the genes/loci related to end-use quality traits in wheat (Guo J. et al., 2020). The QTLs identified from the mapping studies are first located in the physical map of the chromosome and the candidate genes located in the QTL are noted using the gene annotation available from the assembly (Li Y et al., 2012). This has allowed the identification of novel QTLs/genes related to end-use quality traits in wheat (Li Y et al., 2012). Based on the gene function annotation and existing knowledge of quality development metabolic pathways, the function of these candidate genes are classified (Yang et al., 2020). Fine mapping has also been done to narrow down the list of candidate genes or to even identify the causal gene for traits such as GPC, grain weight and size in wheat (Olmos et al., 2003; Distelfeld et al., 2006; Uauy et al., 2006; Röder et al., 2008; Zhang et al., 2018). Markers related to the gene can then be used for marker-assisted selection of individuals having the desired genotype of interest. Wheat genome assembly has also allowed study of the homologs of the genes that could be present in all three subgenomes of wheat i.e., A, B and D genome. Moreover, it also allows assessments of intraspecies genomic variation and the availability of multiple assemblies from different lines can be further used for comparative mapping (Walkowiak et al., 2020).

All these genetic and genomic studies in wheat have generated valuable resources for wheat including reference genomes, whole-genome sequencing data, shotgun sequencing data, genome-wide genetic marker data, gene expression atlas, and diagnostic markers which are available across various platforms. Genome reference sequence browsers are available for different cultivars/species of wheat including the Chinese spring wheat (Appels et al., 2018), HRWW varieties such as Jagger and Mace, spelt, durum wheat, and wild relatives of wheat such as wild emmer and *Ae. tauschii* (https://wheat.pw.usda.gov/GG3/genome_browser#). PlanGDB (http://www.plantgdb.org/), Gramene (http://www.gramene.org/), and Phytozome (https://phytozome.jgi.doe.gov/pz/portal.html#) are additional sources for genomic information on many crops including wheat. In 2019, an international collaboration project “10 + genome project” released a wheat pan-genome containing a reference sequence of 10 global panels of wheat varieties (http://www.10wheatgenomes.com). As of November 2022, sequencing data from more than 61,500 bio-samples related to common wheat have been deposited in NCBI and are publicly available (https://www.ncbi.nlm.nih.gov/sra). These resources are being used for advancing studies of end-use quality traits in wheat and will continue to be useful in the future.

## 6 QTL/genes related to end-use quality traits in wheat

The genetic, genomic, and physiological information for various end-quality traits in wheat continues to grow. This includes the identification of major QTLs/genes controlling various traits of interest. These results will be instrumental for the continued success of GAB in wheat. The following are some of the major findings for end-use quality traits in wheat.

### 6.1 Grain protein content (GPC)

Joppa et al. (1997) identified a significant QTL related to GPC on chromosome 6BS contributing up to 66% phenotypic variance (PV) from a population of RIL of T. turgidium L. var dicoccoides (Table 1). This QTL was later mapped as a single Mendelian locus, Gpc-6B1, flanked by Xcdo365 and xUCW67 markers at 1.5 cM and 1.2 cM, respectively (Olmos et al., 2003). Distelfeld et al. (2006) carried out microcolinearity between the rice reference genome and wheat expressed sequence tags (ESTs) which narrowed down the position of the Gpc-B1 gene to .3 cM flanked by PCR markers Xucw79 and Xucw71. The same authors also identified a more tightly linked (.1 cM) high throughput codominant marker Xuhw89 that was suggested for the initial screening of the Gpc-B1 gene. So far, many QTLs have been identified for GPC, however improvement in GPC of wheat has been limited to the introgression of this major gene, *Gpc-B1* (Sengar and Singh, 2018). Another important finding for GPC was done by Terasawa et al. (2016) who studied DH lines of HRWW and identified a single major QTL, *QGpc.2B-yume*, on chromosome 2BS that explained 32% of PV for GPC and 16.5% of PV for FPC. The authors also recommended the use of flanking SSR marker *Xgpw4382* for MAS of the identified QTL. Similarly, Kumar A et al. (2019) studied GPC in RIL of common wheat where they identified two major and stable QTLs, *QGPC. ndsu.7A.2* and *QGPC. ndsu.7B*, in chromosome 7AL and 7B that explained 14.6% and 24.9% of PV, respectively. Additional studies on GPC have been summarized in Table 1.

### 6.2 Glutenin and gliadin

In hexaploid wheat, HMW-GS is encoded by three loci, Glu-A1, Glu-B1, and Glu-D1, found on the distal half of the long arm of chromosomes 1A, 1B, and 1D, respectively, whereas the LMW-GS loci, *Glu-A2*, *Glu-B3*, *Glu-D3* are found on short arms of those same group 1 chromosomes (Payne, 1987; Singh and Shepherd 1988). HMW-GS are extensively studied in wheat as they play a major role in dough elasticity (Payne, 1987). Wheat varieties can contain tightly linked genes with multiple alleles within these loci that code for “x” and “y” type glutenin subunits (Payne et al., 1981; Payne, 1987; Ravel et al., 2006; Mann et al., 2009). Ravel et al. (2020) identified 8, 22 and 9 different alleles at *Glu-A1*, *Glu-B1* and *Glu-D1* locus, respectively using the sodium dodecyl sulfate–polyacrylamide gel-electrophoresis (SDS–PAGE) method. The subunit “Dx5+Dy10” coded by Glu-D1d, “Ax2” coded by Glu-A1b, and “Bx7+Bx8” coded by Glu-B1b have been found to have a positive effect on dough properties resulting in good bread making quality (Payne and Lawrence 1983; Shewry 2003; Pirozi et al., 2008; Ravel et al., 2020). Ravel et al. (2006) carried out an association study in bread wheat and identified Glu-B1-1 as the candidate gene for determining the quantity of HMW glutenin. Payne et al. (1984) identified the association of gluten strength and LMW glutenin subunits (LMW-GS), and the gliadins were found to be tightly linked to the LMW-GS. The α and β gliadin genes are found on the short arms of chromosomes 6A, 6B, and 6D, whereas the γ and ω gliadins occur in the same locations as the LMW glutenin subunits (Payne et al., 1984; Payne, 1987).

### 6.3 Starch

There are three homoeologous waxy genes in common wheat, Wx-A1, Wx-B1, and Wx-D1, located on chromosomes 7AS, 4AL, and 7DS, respectively (Chao et al., 1989), that produce three distinct Wx proteins (Nakamura et al., 1993). Chao et al. (1989) indicated that the Waxy gene originally present on chromosome 7B was translocated to chromosome 4AL. Based on the presence/absence of Wx proteins, wheat plants were categorized into eight groups: Type 1 for wild type (having all Wx-A1, Wx-B1, and Wx-D1 proteins), Type 8 for waxy wheat (no Wx protein), and Type 2–7 for partial waxy lines (with missing one or two Wx proteins) (Nakamura et al., 2002). Nakamura et al. (1993) developed the first waxy tetraploid (amylose free) by crossing partially waxy plants, Type7 common wheat and Type IV durum. Such waxy wheat has been used to products such as Asian wet noodles (Graybosch, 1998). Guzmán and Alvarez (2016) reported that there are 19 different waxy protein variants, and so far, molecular studies have identified 19, 15, and 7 allelic variants for Wx-A1, Wx-B1, and Wx-D1 gene, respectively. The *Wx-A1a*, *Wx-B1a* and *Wx-D1*a are the wild type alleles for these genes (Yamamori et al., 1994; Nakamura et al., 1995). Apart from normal functional alleles, alleles with loss of function (i.e., null allele) have also been identified for all three genes with null alleles for *Wx-A1* and *Wx-B1* gene being more common than *Wx-D1 gene* (Guzmán et al., 2012b). The use of null alleles allows researchers to create partial waxy and waxy starch (Zhang et al., 2022), that has a beneficial effect on quality of products as discussed above. Therefore, identification and discovery of the alleles has been of major focus for grain starch. The *Wx* genes consist of 12 exons and 11 introns similar to that in barley and molecular markers have been successfully used to identify null mutants for these genes (Guzmán and Alvarez, 2016).

### 6.4 Grain hardness

Early studies (Symes, 1965) speculated that the grain hardness that distinguishes between hard and soft wheat is caused by a single major gene. This Hardness (Ha) locus is present in the short arm of chromosome 5D and contains Pina-D1, Pinb-D1, and Gsp-1 genes which encode for puroindolines a (Pina), puroindolines b (Pinb), and Grain Softness protein-1, respectively (Morris, 2002; Bhave and Morris, 2008; Guzmán and Alvarez, 2016). So far, nine Pina alleles and 17 Pinb alleles have been identified, out of which Pina-D1b, Pinb-D1b, Pinb-D1c, and Pinb-D1d are the major alleles identified in hard wheat cultivars (Bhave and Morris, 2008; Huang and Brûlé-Babel, 2011).

The durum wheat (genome AABB) has no D genome (Morris et al., 2011) therefore, the *Ha* locus is completely absent and it expresses extremely hard texture, harder than “hard” common wheat (Morris et al., 2011). A novel soft grain texture referred as “super soft” has also been identified among common as well as durum wheat and is found to be associated with higher break flour yield (Morris et al., 2011; Wang et al., 2012; Ibba et al., 2019; Kumar N et al., 2019). Kumar N et al. (2019) identified four major QTLs on chromosome 1BS, 4BS, 5AL and 7AS explaining 15%–19% of phenotypic variance for soft kernel texture in spring wheat. Wang et al. (2012) and Ibba et al. (2019) also identified QTLs associated to grain texture on chromosome 1BS, 4BS, 5BS, 2DS, 4DS and 5DL. These results provide evidence that the grain hardness is also controlled by genomic regions other than the *Ha* locus. The identification of super soft grain texture is also very useful as it could possibly provide bakers a new type of flour (Kumar A et al., 2019). These findings will further help to improve the understanding of kernel texture in common and durum wheats.

### 6.5 Sedimentation volume (SV)

Several studies have identified many loci associated with SV on chromosomes 3A, 3B, 4A, 4B, 5A, 5B, 6A, 7A, and 7B (Blanco et al., 1998; Elouafi et al., 2000; Patil et al., 2009). Patil et al. (2009) identified three main effect QTLs for SV in proximity to Glu-B1, Glu-B2, and Glu-B3 glutenin coding loci. In the same study, the QTL QSv.macs-1B.1, flanked by marker interval Xgwm550 and Glu-B3, explained 9.18%–40.6% of PV across five environments. The same authors also found that 22 main effect QTLs for various quality traits such as SV, GPC, and mixograph parameters formed five different clusters on chromosomes 1B, 4B, 7A, and 7B.

To summarize, genetic mapping studies for end-use quality traits in wheat have provided valuable information regarding putative causal/candidate genes or loci related to these traits. Besides this, many studies have also reported an important physiological correlation between various quality traits in wheat including the negative correlations between GPC and grain yield (Cox et al., 1985; Simmonds, 1995). Yang et al. (2020) discovered that the GPC, wet gluten content, and starch content in wheat to be highly positively correlated. The authors also suggested the use of such pleiotropic QTL for the selection of improved quality traits. A significant positive correlation has also been found between dough rheological properties such as dough development time and dough stability time (Li J et al., 2012; Yang et al., 2020). However, a negative correlation was reported between GPC and dough stability time and grain hardiness, wet gluten content and dough stability time and wet gluten content and dough development time (Li J et al., 2012). This information will be useful for improvement of end-use quality traits in wheat.

## 7 Marker-assisted breeding (MAB) and translational genomics for end-use quality traits

The use of molecular markers has significantly impacted the area of plant breeding and genetics. The linkage between major genes and quantitative trait effects was first reported by Sax (1923) and later Thoday (1961) reported the use of gene markers to locate the QTL. Molecular markers improve the efficiency of breeding programs by allowing early generation screening of individuals for targeted trait(s) which saves/reduces resources, energy, and time (Gale, 2005; Xu and Crouch, 2008). Molecular markers have been utilized for the selection of individuals in a breeding program through MAS and GS. Since quality traits are evaluated on harvested grains, i.e., at the end of the crop season, they are ideal targets for MAS and GS (Hayes et al., 2017).

### 7.1 Marker-assisted selection (MAS)

MAS is an important tool in plant breeding. Molecular markers can either be linked to the gene/locus or are diagnostic for a trait of interest (Gale, 2005; Xu and Crouch 2008). QTL mapping and GWAS studies in wheat have been able to identify markers that are tightly linked to the genes/locus influencing the trait and are therefore co-inherited with the trait. On the other hand, diagnostic/functional markers are those that are developed from actual gene sequence influencing the trait and they do not need independent validation for each parental line in a breeding program (Gale, 2005; Lau et al., 2015). MAS is used in many conditions including: 1) breeding for traits where conventional phenotypic selection is difficult, costly or time-consuming; 2) breeding for traits with high environmental influence or for traits whose selection depends on a specific environment and/or developmental stage; 3) speed breeding backcrossing or maintenance of recessive alleles during backcrossing also referred as marker-assisted backcrossing (MABC); and 4) pyramiding multiple favorable alleles within a single population also referred as marker-assisted recurrent selection (MARS) (Xu and Crouch, 2008).

The use of MAS method for end-use quality traits is a much more convenient, efficient, and faster alternative than the conventional method of phenotypic measurement and selection of individuals. In the past two decades, several studies have identified markers related to quality traits that were recommended for use in MAS. For wheat, there are more than 97 functional markers that are being used in breeding programs for various traits of interest including end-use quality traits (Nadeem et al., 2018). Table 2 provides an overview of markers being used for MAS of five end-use quality traits in wheat.

The PCR markers, Xucw71 and Xuhw89 (codominant), developed by Distelfeld et al. (2006) have been used for selecting genotypes having the Gpc-B1 gene related to high GPC content (https://maswheat.ucdavis.edu/protocols/HGPC) (Table 2). Bokore et al. (2019) also validated the use of Xucw71 and Xuhw89 markers for MAS of genotypes with or without the Gpc-B1 gene from four common hexaploid wheat populations. The authors reported an increment in the GPC content of lines with the Gpc-B1 positive allele. Similarly, Ravel et al. (2006) claimed that the Glu-B1 linked markers can be used by breeders for the indirect selection of high protein genotypes.

MAS has also been used for traits other than GPC in wheat. Huang and Brûlé-Babel (2011) developed simple and co-dominant PCR markers to select grain hardness targeting the *Pina* and *Pinb* alleles (Table 2). Patil et al. (2009) recommended the use of markers *Xgwm550* and *Glu-B3* flanking the QTL *QSv.macs-1B.1* for MAB of SV. Likewise, Ibba et al. (2018) developed haplotype specific molecular markers for identification of specific haplotypes of *Glu-A3*, *Glu-B3* and *Glu-D3* locus associated with gluten strength.

The negative relationship between grain yield and GPC, and the significant interactions between these traits and the environment make it challenging for GPC improvement through conventional breeding (Cox et al., 1985; Simmonds, 1995; Rapp et al., 2018) and MAS has been suggested for improvement in both traits. The negative relationship between these traits could be due to plants requiring more energy to produce the same amount of protein than carbohydrate (De Vries et al., 1974; Blanco et al., 1996). One of the strategy for ameliorating such negative effect is to do the MAS of GPC simultaneously with the phenotypic selection for other yield and quality traits (Tabbita et al., 2017). As an example, Kumar et al. (2011) carried out MAS of *Gpc-B1* and reported that MAS-derived progenies did not show proportional decline of grain yield. Therefore, the authors were able to select for lines with high GPC and no penalty for grain yield. The other alternative would be to identify genes with no negative effect on the grain yield and use it in breeding program. Terasawa et al. (2016) identified a major QTL, *QGpc.2B-yume*, on chromosome 2B which had no significant negative effect on grain yield and other yield components traits. The effect of *QGpc.2B-yume* should be tested in other populations and environments as well. These findings are promising for the development of wheat varieties with improved yield and end-use quality.

Vishwakarma et al. (2014) carried out MABC for introgression of *Gpc-B1* from a high GPC variety, Glu269, to an elite variety HUW468. Elite lines were developed within a relatively short period of two and half years (five crop cycles), and improved lines were selected with significantly higher GPC and consisted of 88.4%–92.3% of the recurrent parent plant genome. The authors utilized the SSR marker *Xucw108* developed by Uauy et al. (2006) for foreground selection, and 86 other polymorphic SSR markers were used for background selection on recovery of the recurrent parent plant genome. Similarly; Rai et al. (2019) carried out MABC for the introgression of the grain softness gene, *PinaD1a*, into a hard-grained variety to develop soft grain wheat lines. In this study, the authors carried out a foreground selection for the *PinaD1a* allele using *PinA* marker (Gautier et al., 1994), background selection using 173 SSR markers covering all 21 chromosomes, and a negative selection for *Pina-D1b* allele using Pina-N1 marker (Chen et al., 2013). MARS has also been used in wheat breeding for grain yield and end-use quality traits. Maich et al. (2020) reported results from 12 cycles (24 years) of recurrent selection for grain yield among 83 F1 hybrids obtained by crossing 16 commercial varieties. The authors obtained a yield increment of 1.3% per year and at the end of 12 cycles and the improvement in yield did not affect the baking quality of wheat.

Although MAS seems a promising step forward in the field of MAB, it does have some limitations. MAS is based on the prediction accuracy of previously identified significant markers linked to major traits and these markers are few in number (Heffner et al., 2011). This limits the use of MAS for complex quantitative traits as accurate prediction of these traits will require markers for all of the associated genes (Heffner et al., 2011). These genes/QTLs also interact with each other at different expression levels thus changing the phenotype of an individual. MAS cannot account for these interaction effects because most of the markers are initially developed from a mapping population segregating for a single or few QTL which usually leads to overestimation of the QTL effect. Therefore, MAS is mostly constrained to simply inherited or monogenic traits (Michel et al., 2018). In addition, MAS also does not factor the environmental influence on the trait leading to lower phenotype prediction accuracy (Xu and Crouch, 2008; Heffner et al., 2009). Breeding programs now have integrated GS in their MAB as it considers all of these limitations of MAS, and these two tools complement each other.

### 7.2 Genomic selection (GS)

In GS, individuals are selected based on whole-genome marker profiling data that provides an overall performance evaluation of the plant (Varshney et al., 2015). The use of a whole-genome marker increases the chance that all QTLs are in linkage disequilibrium with at least one marker (Varshney et al., 2015). Due to this reason, unlike MAS, the GS does not require prior knowledge about large effect QTLs (Guo Y. et al., 2020). Also, for quantitative traits, GS usually has higher prediction accuracy than conventional MAS. In GS, phenotypic and genotypic marker data from the training population are fitted into the statistical models to get an estimation of all marker effects which are then used on unobserved genotypes of a testing population to calculate the genomic estimated breeding value (GEBV) (Heffner et al., 2009). Individuals are then selected based on the GEBV. The training population must be representative of the testing population to maximize the GEBV accuracy (Heffner et al., 2009). These training populations are advanced breeding materials that have been well characterized phenotypically and the GEBV of the testing population genotypes is predicted based on their genetic relation with the training population (Guzman et al., 2016; Michel et al., 2017; Michel et al., 2018). To assure proper selection, well-known check varieties, such as varieties with both good and poor bread-making quality, are included in the GS process that will help in validation of the selection as well as provide a reference for quality profiles (Guzman et al., 2016). However, extensive and good phenotypic data of the training population is required at the forefront in the wheat breeding program to generate accurate GEBV before GS can be used to select quantitative traits.

GS allows a 2–3 years earlier selection of many end-use quality traits in wheat than traditional selections and it is done on a much broader population allowing the selection of lines with good end-use quality and higher yield (Michel et al., 2018). Table 3 summarizes some of the GS studies carried out over the last decade for end-use quality traits in wheat. CIMMYT has been conducting GS for end-use quality traits in wheat since 2012 (Battenfield et al., 2016). In the CIMMYT breeding program, ∼10,000 first-year yield trials lines are genotyped and GEBV values are estimated for grain yield and end-use quality traits (Guzman et al., 2016). Heffner et al. (2011) compared MAS, GS, and phenotypic prediction accuracy of genetic value for nine different grain quality traits in biparental populations of soft winter wheat and found that the GS to phenotypic selection accuracy was as high as .66 and the GS prediction accuracy was superior to conventional MAS (Table 3). Similarly, Hayes et al. (2017) conducted a study on genomic prediction of 19 end-use quality traits and observed prediction accuracy greater than .5 for many of the traits (Table 3). Besides this, Michel et al. (2018) also carried out GS of baking quality in wheat and reported that an acceptable prediction accuracy of .38–.63 can be obtained in all dough rheological traits.

GS is still emerging, and advanced computation models are being developed to increase the accuracy in the prediction of GEBV value. Several GS models have been developed which also account for G×E interaction thus increasing prediction accuracy across environments (Heslot et al., 2014; Jarquín et al., 2014). Known candidate genes can be used to correlate the genotype-level and gene-level G×E interactions which can be used to predict the influence of G×E interaction (Li et al., 2018). Models are being developed for various G×E conditions including: 1) predicting tested genotypes in untested environments; 2) predicting untested genotypes in tested environments, and 3) predicting untested genotypes in untested environments (Li et al., 2018). Multi-trait GS models have also been developed that can account for the genetic correlation among the traits and improve the prediction accuracy of the primary trait when data for secondary correlated traits are available (Gill et al., 2021). Sandhu et al. (2022) carried out genomic prediction for seven end-use quality traits in winter wheat and found that the multi-trait models performed 5.5% and 7.9% superior to uni-trait GS models for within-environment and across location predictions, respectively, and multi-trait-multi-environment models performed 10.5% superior to the uni-trait models. Similar results have been found in other GS studies in wheat as well (Hayes et al., 2017; Guo J. et al., 2020; Gill et al., 2021). These results will certainly increase breeder’s confidence in integrating GS into their breeding program.

The UGA and SUNGRAINS (Southern Universities Grains, http://www.sungrains.lsu.edu/data.shtml), a cooperative research program between seven universities has been utilizing GAB for the development of superior germplasm of SRWW for the southeast United States. In collaboration with the USDA-ARS genotyping center (lead by Dr. Gina Brown-Guedira, https://wheat.pw.usda.gov/GenotypingLabs/?q=about), UGA and the SUNGRAINS breeding programs are using GS for important economic traits since 2015. MAS is also an important part of the program. Diagnostic markers are used for the selection of desirable genotypes for traits such as grain texture/softness using *Pinb-D1a* and *Pinb-D1a* markers, GPC using *Gpc-B1* marker, and HMW-GS using *Glu-A1* (Ax2, Ax1, and Axnull subunits), *Glu-B1* (Bx7^OE^), and *Glu-D1* (Dx5+Dx10, Dx2+Dx12) markers etc.

### 7.3 Candidate gene approach (CGA)

CGA is one of the translational genomics tools that has been practiced in wheat for different traits including thousand kernel weight (TKW), kernel size, GPC, yield, plant height, and disease resistance (Faris et al., 1999; Zhang et al., 2014; Nigro et al., 2019; Sehgal et al., 2019; Zhang et al., 2020). Zhang et al. (2014) carried out a translational genomics study of grain size regulation gene in wheat where they utilized the rice candidate gene, OsGS3. This gene is well characterized, negative regulator of grain size and explained 80%–90% of phenotypic variation for grain weight and grain length in rice. The orthologous TaGS gene in wheat, *TaGS-D1* on chromosome 7DS was identified based on sequence identity and predicted protein similarity (Zhang et al., 2014). The authors were also able to identify QTL associated with *TaGS-D1* using SSR markers for chromosome 7D. Similarly, a recent study by Nigro et al. (2019) utilized 14 candidate genes for GPC and/or yield for gene-based association mapping by identification of SNP for candidate genes across a diversity panel. The authors found that genes, *AlaAT4A*, *ASN1-5A*, *NR-6A*, and *GS2-2B* were significantly associated with GPC across seven environments. They further identified 11 stable QTL for GPC using GWAS and suggested that the utilization of the CGA and GWAS in parallel can increase the power and precision of QTL detection by reducing Type I and Type II error rates. Garg et al. (2009) identified a novel pair of HMW glutenin subunits, *Glu-S* ^*s*^ *1* from a study on Ae. searsii, an S genome wild ancestor of wheat. They indicated that these would be good candidate subunits that can improve the bread-making quality of wheat.

## 8 Challenges and future opportunities for improving wheat end-use qualities

Phenotyping for end-use quality traits is challenging and requires a great deal of resources and time, owing to the fact that the quality assessment for direct screening of elite breeding lines is only feasible later in the advanced generations when other important traits including grain yield, disease and pest resistance have been selected for. Therefore, it is imperative to optimize phenotyping and selection of end-use quality traits. An effective strategy is to study the G×E interaction for end-use quality traits. Traits highly influenced by environment, such as GPC and FPC, should be tested in multiple environments to allow breeders to get a better estimation of the value of a particular genotype (Aoun et al., 2021). Traits not as affected by environment can be tested in fewer environments which saves resources to test additional lines in the breeding program (Aoun et al., 2021). In addition, the G×E interaction provides information on genotype stability across environment and identifies the target environment for maximizing genetic gain (Dias et al., 2018). Moreover, future advancements in phenotypic analysis equipment and rapid assay techniques, including automated phenotyping and phenomics, could further reduce time to analyze multiple samples and reduce resources.

Not all research facilities and breeding programs have cutting-edge laboratories, equipment, and expertise to operate and assess all quality attributes. In fact, only a few U.S. states and federal agencies (e.g., USDA-ARS) have wheat end-use quality assessment labs available. With regards to such traditional assessment methods, early generation MAS of desirable genotype(s) for the end-use quality traits is much more convenient and efficient. However, available markers are limited to only some of the genes identified so far and are already being used for selecting associated genes in the breeding lines. Therefore, there is a continual need for introgression of other major genes associated with these traits. One of the significant bottlenecks to QTL mapping and identification is the lack of validation studies. Hence, the plethora of QTLs identified to date for several end-use quality traits have few practical implications. Kiszonas and Morris, (2017) stated: “wheat breeding community have collected numerous markers and QTLs, but very few of them are being used for the improvement of wheat …. collecting countless QTLs is rather meaningless unless we take the next step and develop and use markers to improve the quality and consistency of wheat.” Therefore, the wheat breeding community should focus fine mapping on identified, important major QTLs, functionally validate and clone the candidate gene(s), and develop markers associated with the genes for MAS of end-use quality traits. However, it is also important to consider that most QTLs identified so far are not major and/or they explain small portion of PV and are population and/or environment specific. Minor QTLs can be useful for their additive effect, but it is obvious that the major QTLs such as *GPC-B1* are the ones that add the most value in a breeding program. Therefore, the quest for the major/novel QTL(s)/gene(s) should continue in balance with validation studies. Moreover, the identified major genes can also be integrated as a fixed effect in the genomic prediction models for further improving the accuracy of GS (Bernardo, 2014).

Conventional genetic map-based QTL/gene discovery of traits requires screening of numerous breeding lines to identify the individuals segregating for the traits of interest. However, individuals segregating for traits of interest may not be present in the germplasm managed by the breeding program or the identified loci for segregating population could only explain a small amount of phenotypic variance. Besides this, the development of crosses could also be troublesome in the plants due to flower morphology or unsynchronized flowering between the lines. Therefore, researchers instead can look for studies done on the same traits across crops or even across related plant species to find genomic information that can be translated to the target crop, wheat. For example, wild emmer, *T. turgidum* L ssp *dicoccoides*, contains higher GPC than most hexaploid/tetraploid wheat cultivars (Avivi, 1978; Nevo et al., 2002) and could potentially be used to identify positive alleles of genes governing the GPC (Distelfeld et al., 2006). Therefore, studies done on GPC in wild emmer can provide useful genomic information to improve the GPC of durum and hexaploid wheat using the methods discussed above. In addition, other crop species within the Gramineae family such as rice, maize, barley, and oat have a greater chance of synteny or gene co-linearity with wheat and can influence similar phenotypes. Therefore, genetic findings on end-use quality traits in these crops should be further explored for translation to wheat.

Translational genomics through comparative genomics and functional genomics allows for a more integrated improvement of crop species. Non-etheless, translational genomics can be challenging due to the limitations of the molecular breeding approaches and tools used by the breeding programs. The use of MAS could be hindered if there are limited significant markers for the traits of interest. The GS can be limited based on the models used for the prediction of breeding values and the density of the markers. The CGA approach is solely based on the identification of functionally characterized genes or positional candidate genes which can be limited in number. However, these challenges can be overcome as additional research and findings are being put forth requiring continued and improved access to data. Therefore, it is necessary for the development of a single database that allows access to all these resources as proposed by (Kang et al., 2016). Infrastructure and management system of these data and the competence to handle large/sophisticated data are critical (Varshney et al., 2015). Additionally, the future of translational genomics also depends on how accessible these NGS technologies are to breeding/research programs. Genomic information and breakthroughs are the foundations for translational genomics for crop improvement in the future. This would also be beneficial to neglected and underutilized, yet nutritionally important crops that are being left behind from genetic improvement such as quinoa, finger millet etc. (Subedi, 2020; Gautam et al., 2021).

Consideration should also be given for the establishment of expanded private-public research collaborations which have been in limited number and scale in wheat breeding. These two institutions worked independently with a common goal of developing improved varieties of wheat. As a result, these two institutions were in competing space with each other. Therefore, there is a need for establishment of a collaboration where both benefit without compromising the institutional integrity and success. Such collaboration could be made by sharing resources such as epidemiological information, technologies such as sequencing platforms, labs, equipment such as planters and harvesters or research fields for testing varieties in diverse environmental conditions. One such example is the International Wheat Yield Partnership (IWYP) (https://iwyp.org/) where both private and public institutions are working together to achieve a common goal of increasing the genetic yield potential of wheat by 50% by 2035 (https://iwyp.org/global-challenge/). The partnership combines the discovery, and research expertise of the public institutes with the private industries excellence in taking validated discoveries into breeding programs, developing putative varieties, large-scale testing, multiplying seeds of the varieties and delivery of improved varieties to farmers across the globe. There is a need of such bilateral relationship between universities and industries and the joint findings can be further disseminated in collaboration with the extension service within each of the institutions.

To conclude, research towards improved end-use quality of wheat utilizing recently developed modern tools/methodologies and technologies such as MAS, GS, and CGA clearly indicate that the adoption of GAB is increasing and will likely continue to expand in the future. This approach facilitates the plant breeding objectives of identifying and selecting individuals with desirable and economic end-use quality traits in a more effective and efficient way. It speeds up the process of crop improvement and the development of adapted cultivars. Utilization of GAB approaches, combined with targeted phenotypic assessments to validate plant selections, provides wheat breeders with ever-powerful tools to maximize wheat improvement. It is also vital to innovate ways that allow both private and public sectors to work hand-in-hand to address future challenges, including increasing productivity and end-use quality of major crops. Overall, understanding and modifying the crop genomic architecture will advance wheat germplasm with promising end-use quality traits in addition to increased grain yield that can ensure food and nutritional security for the escalating global population.
